# Interleukin-Encoding Adenoviral Vectors as Genetic Adjuvant for Vaccination against Retroviral Infection

**DOI:** 10.1371/journal.pone.0082528

**Published:** 2013-12-04

**Authors:** Inga Ohs, Sonja Windmann, Oliver Wildner, Ulf Dittmer, Wibke Bayer

**Affiliations:** 1 Institute for Virology, University Hospital Essen, University Duisburg-Essen, Essen, Germany; 2 Division of Pharmacovigilance, Paul-Ehrlich-Institut, Langen, Germany; University of South Carolina School of Medicine, United States of America

## Abstract

Interleukins (IL) are cytokines with stimulatory and modulatory functions in the immune system. In this study, we have chosen interleukins which are involved in the enhancement of T_H_2 responses and B cell functions to analyze their potential to improve a prophylactic adenovirus-based anti-retroviral vaccine with regard to antibody and virus-specific CD4^+^ T cell responses. Mice were vaccinated with an adenoviral vector which encodes and displays the Friend Virus (FV) surface envelope protein gp70 (Ad.pIXgp70) in combination with adenoviral vectors encoding the interleukins IL4, IL5, IL6, IL7 or IL23. Co-application of Ad.pIXgp70 with Ad.IL5, Ad.IL6 or Ad.IL23 resulted in improved protection with high control over FV-induced splenomegaly and reduced viral loads. Mice co-immunized with adenoviral vectors encoding IL5 or IL23 showed increased neutralizing antibody responses while mice co-immunized with Ad.IL6 or Ad.IL23 showed improved FV-specific CD4^+^ T cell responses compared to mice immunized with Ad.pIXgp70 alone. We show that the co-application of adenoviral vectors encoding specific interleukins is suitable to improve the vaccination efficacy of an anti-retroviral vaccine. Improved protection correlated with improved CD4^+^ T cell responses and especially with higher neutralizing antibody titers. The co-application of selected interleukin-encoding adenoviral vectors is a valuable tool for vaccination with regard to enhancement of antibody mediated immunity.

## Introduction

Since the discovery of HIV in the early 1980s, the development of a safe and efficient vaccine has been an unmet challenge. The use of attenuated virus for immunization was soon recognized to be too dangerous to be considered in clinical settings (reviewed in 1), and protein vaccines were found to be ineffective [2]. Hence, for the first time in vaccine research, efforts became focussed on the development of an efficient gene-based vaccine. Until now, despite the evaluation of numerous vaccine candidates in preclinical models, only few vaccines were tested in phase IIb or III clinical trials. In one phase III trial a protein-based vaccine based on recombinant HIV Env gp120 was tested, but it was found to confer no protection [2]. In the phase IIb STEP trial, adenoviral vectors encoding HIV Gag, Pol and Nef were used to induce cell-mediated immune responses, but the trial was halted prematurely when no protection from HIV infection or reduction of set-point viral loads was achieved [3]. Very recently, another phase IIb trial, HVTN505, was also halted prematurely due to ineffectiveness. Here, an adenovirus-based vaccine was combined with DNA immunization, and HIV Gag, Pol and Nef as well as Env were used as vaccine antigens to induce cellular as well as humoral immune responses. However, the immunization was found not to decrease HIV incidence or set-point viral loads [4]. Only in the phase III RV144 Thai vaccination trial in a low-risk study cohort, the combination of prime immunizations with canarypox vectors encoding HIV Env, Gag and protease with protein boost immunizations with Env gp120 resulted in moderate protection [5]. This data, and data from animal models [6,7], show that a protective vaccine against HIV infection must be carefully tailored and should induce not only cellular but also strong humoral immune responses. Passive transfer of antibodies has been shown to confer protection of non-human primates from subsequent SIV challenge [8–11], underlining their protective potential. Levels of antibodies induced by vector-based immunization usually do not reach high enough levels to confer strong protection on their own, therefore we focused here on improving the antibody response to adenovirus-based immunization.

In the study presented here we analyzed the adjuvant effect of selected interleukins on immunity against a retrovirus infection conferred by adenovirus-based immunization. Interleukins are major mediators and regulators of the immune system. Interleukins are involved in activation of T cells by antigen-presenting cells, and T cells in turn produce interleukins that act in an autocrine, paracrine or even endocrine manner [12]. Specific interleukins determine the type of CD4^+^ T helper cell (T_H_) response, and specific interleukins are characteristic for the CD4^+^ T_H_ response types and determine the induction of a CTL- versus antibody-dominated immune response. An essential cytokine for the development of T helper type 2 (T_H_2) CD4^+^ cells from T_H_0 CD4^+^ cells is interleukin 4 (IL4) (reviewed in 13), which drives T_H_2 development and at the same time suppresses T_H_1 development. Among the cytokines produced by T_H_2 CD4^+^ cells are IL4, IL5 and IL6, which mediate proliferation and maturation of B cells into plasma cells [14,15]. Therefore, these three interleukins were selected to be used as genetic adjuvants for the improvement of antibody induction by adenovirus-based immunization. Furthermore, we selected the interleukins IL7 and IL23 to be tested as genetic adjuvants. IL7 is mainly known for its role in B cell maturation, and it has also been described to be involved in promoting proliferation and viability of T cells [16]. IL23 belongs to the IL12 cytokine family, it is known to stimulate proliferation of memory CD4^+^ T cells [17], which in turn is important for augmenting and sustaining B cell responses.

To test the adjuvant effect of interleukin-encoding vectors on adenovirus-based immunization, we used the Friend Virus (FV) infection of mice as a model system. FV is a murine retroviral complex consisting of the Friend murine leukemia virus (F‑MuLV) which is replication-competent but apathogenic and the spleen focus forming virus (SFFV) which is pathogenic but replication-deficient. In susceptible mice, FV infection leads to splenomegaly and lethal erythroleukemia [18]. The FV infection of mice is a good model system for the investigation of retroviral infections as well as for the development of anti-retroviral vaccines. Similarly to HIV, complex immune responses including CD4^+^ and CD8^+^ T cell and antibody responses are required for complete protection from FV infection [6]. We have established the FV infection of mice as a useful model for the development of improved adenovirus-based vaccination strategies, where we demonstrated the suitability of specific type I interferons or chemokines to improve adenovirus vector-mediated immunity when used as genetic adjuvants [19,20]. Another step towards improved adenovirus-based immunization was the design of a new type of adenoviral expression/display vector. The special feature of this vector is that the FV antigen is not only encoded by the vector but also displayed on the adenoviral capsid through fusion to the capsid protein pIX. Immunization with this vector induces good protection from FV challenge infection with strong CD4^+^ T cell and binding antibody responses, but significant levels of neutralizing antibodies only become detectable after FV infection [21]. 

Using the adenoviral expression/display vector Ad.pIXgp70 in combination with adenoviral vectors encoding different murine interleukins, we aimed to further improve the vaccination efficiency of this vector especially with regard to the induction of neutralizing antibodies. Therefore, the interleukins IL4, IL5, IL6, IL7 or IL23 were co-delivered by adenoviral vectors as genetic adjuvants to stimulate T_H_2 responses or to directly augment B cell maturation in order to enhance antibody-mediated protection from retrovirus challenge.

## Results

### Co-administration of specific interleukin-encoding adenoviral vectors led to enhanced protection from FV induced splenomegaly

We generated Ad5-based vectors with wild-type or chimeric Ad5/35 fiber (Ad5F35) which are deleted in the E1 and E3 region and encode the murine interleukins IL4, IL5, IL6, IL7 or IL23 (Figure S1). Sequencing and specific ELISAs verified the identity of the encoded interleukins (data not shown). The expression/display vector Ad.pIXgp70 encodes the Friend murine leukemia virus envelope protein gp70 fused to the adenoviral capsid protein pIX (Figure S1) and thus displays gp70 on the capsid, as described previously [21]. 

Highly susceptible CB6F1 mice were immunized twice, first with 1x10^9^ viral particles (vp) Ad5.pIXgp70 and the same amount of interleukin-encoding Ad5 vectors and 4 weeks later in the same way with Ad5F35 vectors (see Figure S2 for a schematic representation of the experimental outline). We demonstrated before that this heterologous prime-boost combination leads to better immunization outcome than the homologous prime-boost immunization with either vector type [22]. As a control, one group of mice received Ad.pIXgp70 in combination with a GFP-encoding or an empty vector to deliver an equal amount of adenoviral vectors to all groups of mice. 3 weeks after the second immunization, mice were challenged with a high dose of 2 500 spleen focus forming units (SFFU) FV. In previous experiments mice were challenged with an infectious dose of 500 SFFU, and immunization with 5x10^9^ vp Ad.pIXgp70 alone led to very good protection from FV-induced disease after this low-dose challenge [21]. However, aiming to further improve the vaccine efficacy, the challenge dose was increased, and the Ad vector dose was decreased, to avoid strong protection by vaccination with Ad.pIXgp70 alone. This allowed us to analyze potential adjuvant effects of immunostimulatory interleukin-encoding adenoviral vectors. 

**Figure 1:**
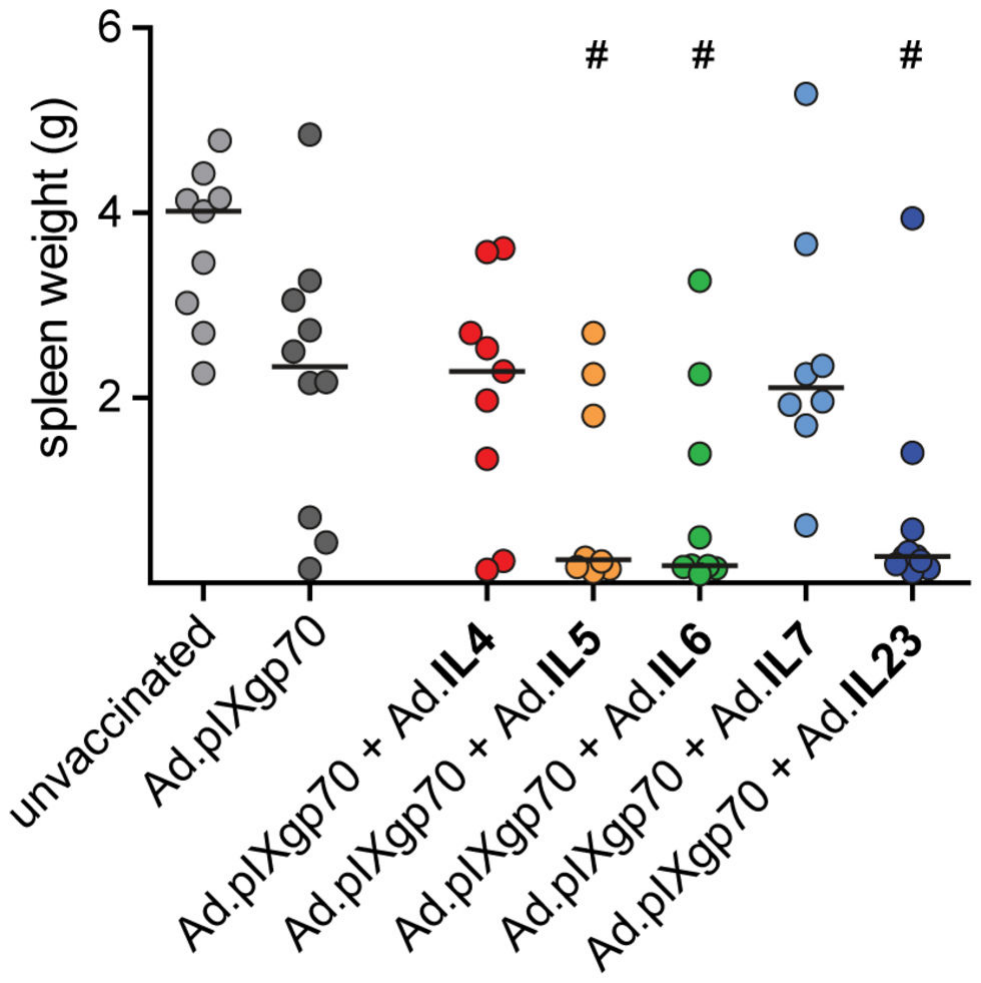
FV-induced splenomegaly in mice immunized with adenoviral vectors. CB6F1 mice were immunized twice with Ad.pIXgp70 in combination with adenoviral vectors encoding specific interleukins. Mice were immunized first with Ad5 vectors and after 4 weeks, mice were boost-immunized with Ad5F35 vectors. Mice of the group Ad.pIXgp70 received in addition a GFP-encoding or empty adenoviral vector so that the total amount of adenoviral particles in all vaccinated groups was the same. Three weeks after the second immunization, mice were challenged with a high dose of FV and spleens were removed and weighed 3 weeks after challenge infection. Statistically significant differences (*P* < 0.05; Kruskal-Wallis one-way analysis of variance on ranks with Dunns multiple comparison procedure) compared to unvaccinated control mice (#) are indicated. Each dot shows an individual mouse, the horizontal lines indicate mean values. Data are results of two independent experiments with similar outcome.

Protection of vaccinated mice from FV-induced splenomegaly was analyzed by weighing of the spleens on day 21 after FV challenge (Figure 1). At this time point, unvaccinated mice had developed massive splenomegaly; the spleen weight of mice immunized with Ad.pIXgp70 alone was not significantly reduced compared to unvaccinated control mice. Co-immunization with vectors encoding IL4 or IL7 did not reduce spleen weights, however mice which were immunized with Ad.pIXgp70 in combination with adenoviral vectors encoding IL5, IL6 or IL23 had significantly smaller spleens than unvaccinated control mice (*P* < 0.05). In addition, the spleens of mice co-immunized with these interleukins were also rather less enlarged than spleens of mice which received Ad.pIXgp70 alone, but this did not reach statistical significance.

In a control experiment, mice were immunized with Ad.IL5, Ad.IL6 or Ad.IL23 alone to exclude any direct effects of the cytokine vectors on subsequent FV challenge infection. No significant differences in spleen weights of unvaccinated mice or mice treated with the interleukin vectors alone were observed 21 days after FV challenge infection (Figure S3A).

### Co-immunization with specific interleukin-encoding vectors mediated improved control over viral replication in vaccinated mice

**Figure 2:**
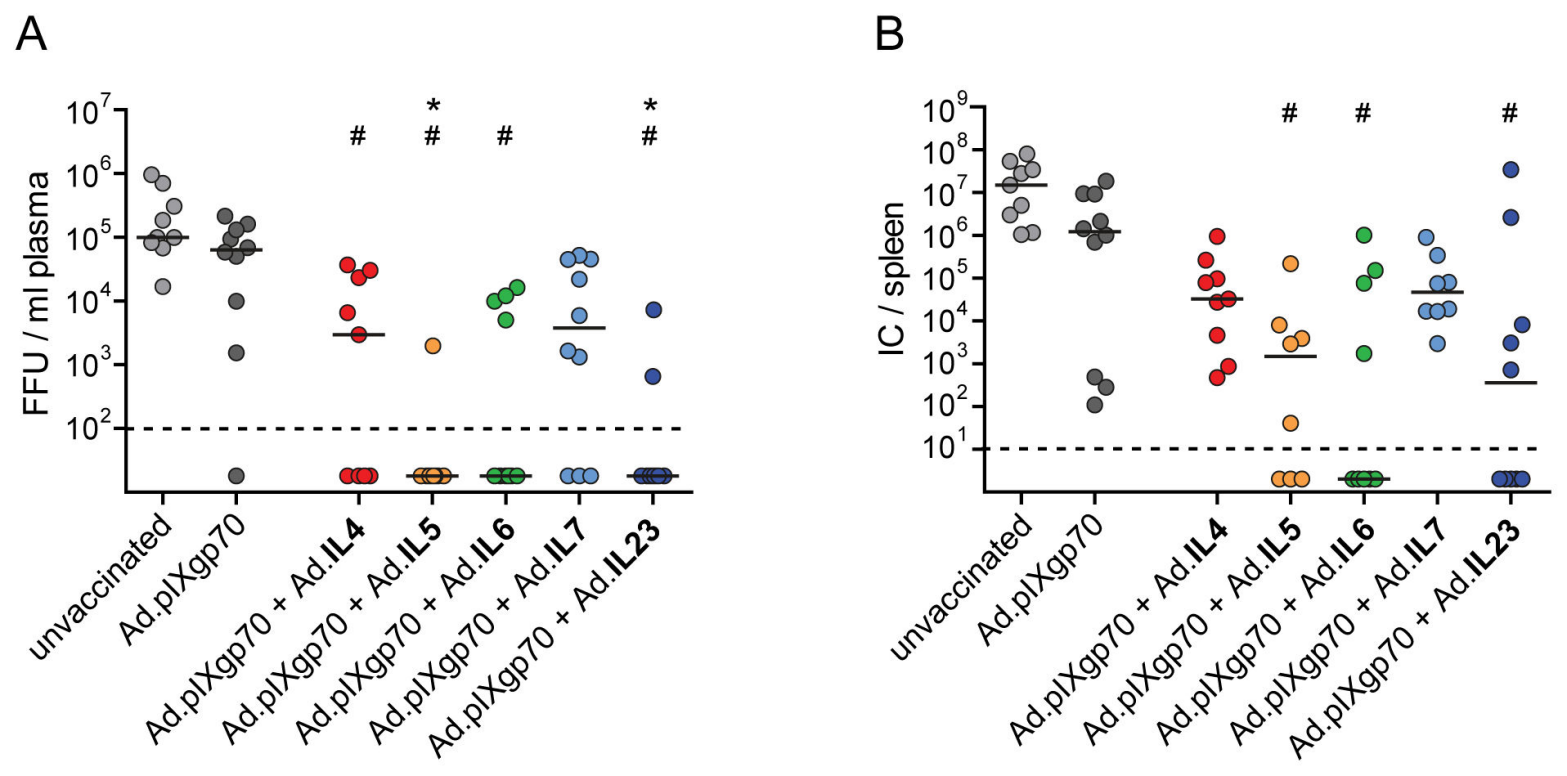
Viral loads of vaccinated mice after FV challenge infection. CB6F1 mice were prime- and boost-immunized with Ad.pIXgp70 in combination with interleukin-encoding adenoviral vectors. Mice were challenged with FV 3 weeks after boost immunization. Plasma viremia (A) in FV infected mice was analyzed on day 10 p.i. and is shown as focus forming units (FFU) / ml plasma, median values are indicated by lines. On day 21 p.i. viral loads in spleen (B) were analyzed and are shown as infectious centers (IC) / spleen, the horizontal lines mark median values. Statistically significant differences (*P* < 0.05; Kruskal-Wallis one-way analysis of variance on ranks with Dunns multiple comparison procedure) compared to unvaccinated mice (#) or mice immunized with Ad.pIXgp70 alone (*) are indicated. For statistical analysis, mice with viral loads below the detection limits were assigned the values 20 for viral load in blood, or 2 for viral load in spleen. Values were subjected to statistical analysis without logarithmic transformation. Each dot represents an individual animal. The dashed lines indicate the detection limits of the assays. Data are results of two independent experiments with similar outcome.

The effect of the co-application of interleukin-encoding adenoviral vectors on the control over viral replication was analyzed on day 10 p.i. (Figure 2A). Vaccination with Ad.pIXgp70 alone did not significantly decrease plasma viremia in comparison to unvaccinated mice, but mice co-immunized with adenoviral vectors encoding IL4, IL5, IL6 or IL23 had significantly lower viremia than unvaccinated mice (*P* < 0.05). The viral loads in plasma of mice which received Ad.IL5 or Ad.IL23 were also significantly lower compared to mice vaccinated with Ad.pIXgp70 alone; in these groups, free virus in plasma was detectable in only 1 out of 9 and 2 out of 10 animals, respectively. Co-immunization with IL7 vectors also resulted in a trend to reduced plasma viremia compared to unvaccinated mice and mice immunized with Ad.pIXgp70 alone, but differences did not reach statistical significance.

In addition to acute viremia levels, numbers of infectious cells in the spleens of vaccinated mice were determined 21 days p.i. (Figure 2B). No significant effects on viral loads in the spleen could be achieved by vaccination with the reduced Ad.pIXgp70 dose alone or by co-application of IL4 or IL7 vectors. In contrast, co-immunization with adenoviral vectors encoding IL5, IL6 or IL23 led to significantly lower viral loads compared to unvaccinated mice (*P* < 0.05). In these groups of mice, virus was undetectable in 3 out of 8, 5 out of 9, and 5 out of 10 mice, respectively.

When mice were immunized with the interleukin vectors alone, no differences in viral loads in plasma on day 10 or in spleen on day 21 after FV challenge were found (Figure S3B&C).

### Increased neutralizing antibody titers after co-administration of specific interleukins

For a protective vaccine against FV, similarly to HIV, the induction of virus-specific antibodies plays an important role [6,23]. We demonstrated before that vaccination with Ad.pIXgp70 alone resulted in good binding antibody titers, but neutralizing antibodies could only be detected after challenge infection [21]. To analyze humoral immune responses we determined total IgG-, as well as IgG1- and IgG2a-subtype binding antibody titers 3 weeks after the first and 2 weeks after the second immunization. After the first immunization, low titers of binding antibodies could be detected in all vaccinated mice, which increased after the boost immunization; however, co-immunization with interleukin-encoding vectors did not improve binding antibody responses compared to immunization with Ad.pIXgp70 alone (data not shown).

**Figure 3:**
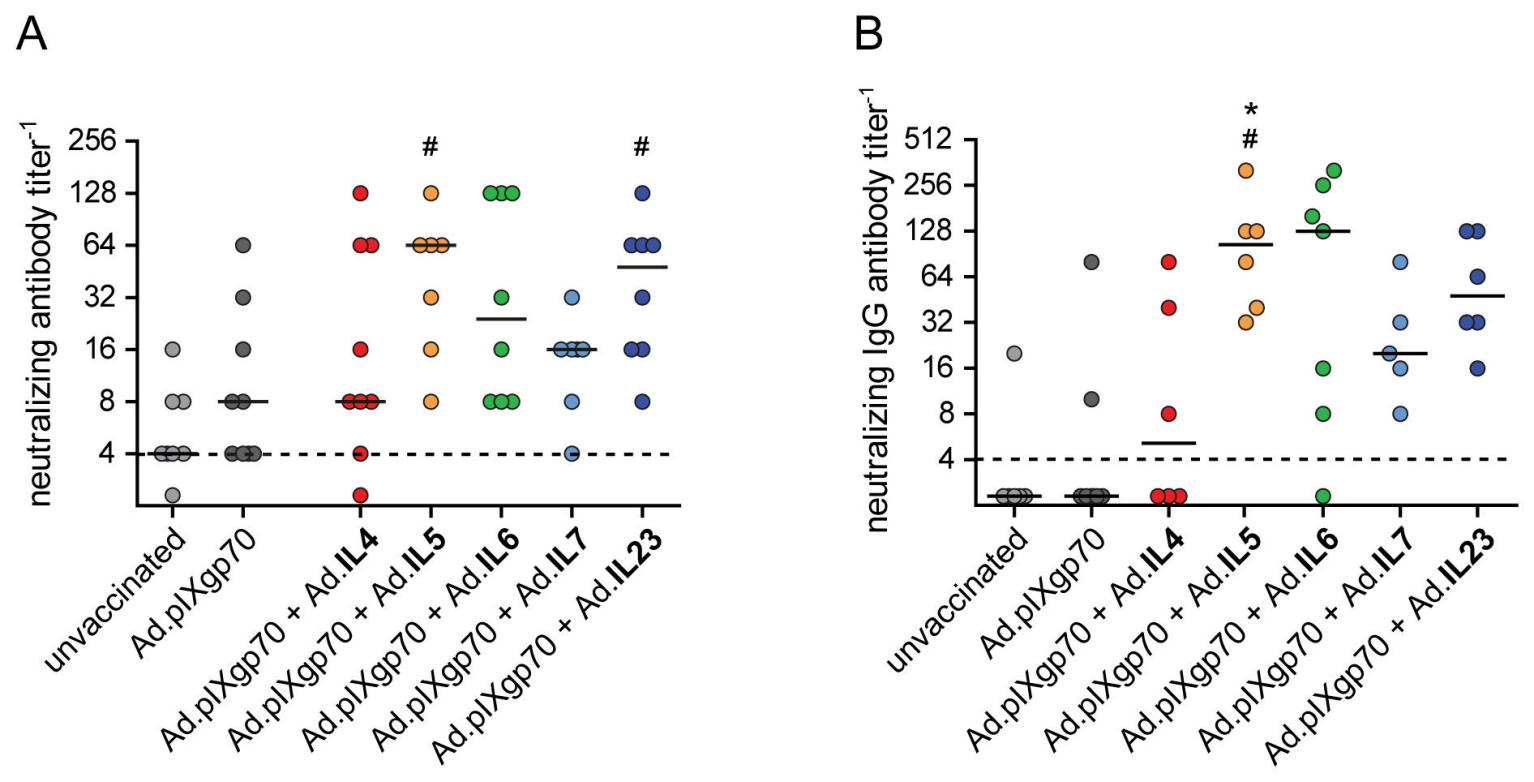
Vaccine-induced FV-neutralizing antibody responses. CB6F1 mice were prime- and boost-immunized with Ad5 and Ad5F35 based vectors of Ad.pIXgp70 combined with interleukin-encoding vectors as indicated. Total neutralizing antibody titers (A) and neutralizing IgG antibody titers (B) were analyzed 10 days after FV challenge infection. Statistically significant differences (*P* < 0.05; Kruskal-Wallis one-way analysis of variance on ranks with Dunns multiple comparison procedure) compared to unvaccinated mice (#) or mice immunized with Ad.pIXgp70 alone (*) are indicated. For statistical analysis, mice with viral loads below the detection limits were assigned the value 2. Values were subjected to statistical analysis without logarithmic transformation. Each dot represents an individual mouse, horizontal lines indicate median values. The dashed lines indicate the detection limit. Data are results of two independent experiments with similar outcome.

For detection of neutralizing antibodies we analyzed blood samples 2 weeks after the second immunization and 10 days after FV challenge infection. As it was seen before, immunization with Ad.pIXgp70 alone induced only low titers of neutralizing antibodies after immunization. The co-administration of interleukin-encoding adenoviral vectors could not significantly improve the neutralizing antibody responses prior to FV challenge infection (data not shown). After FV challenge, mice that had been vaccinated with the reduced dose of Ad.pIXgp70 alone had only low neutralizing antibody titers, whereas mice co-immunized with adenoviral vectors encoding IL5 or IL23 developed significantly higher neutralizing antibody titers compared to unvaccinated control mice ( *P* < 0.05; Figure 3A). The co-administration of IL6 or IL7 vectors also slightly increased neutralizing antibody titers compared to both control groups, but the difference did not reach statistical significance. The difference in neutralizing activity was also apparent when neutralizing antibodies of IgG type were analyzed (Figure 3B), with an obvious trend to enhanced neutralizing IgG antibodies in mice that had received IL5, IL6, or IL23 vectors compared to Ad.pIXgp70 alone. Mice that had been co-immunized with IL5 had significantly higher neutralizing IgG antibody titers than both unvaccinated mice or mice immunized with Ad.pIXgp70 alone (*P* < 0.05).

### CD4^+^ T cell responses were improved by co-administration of select interleukins

**Figure 4:**
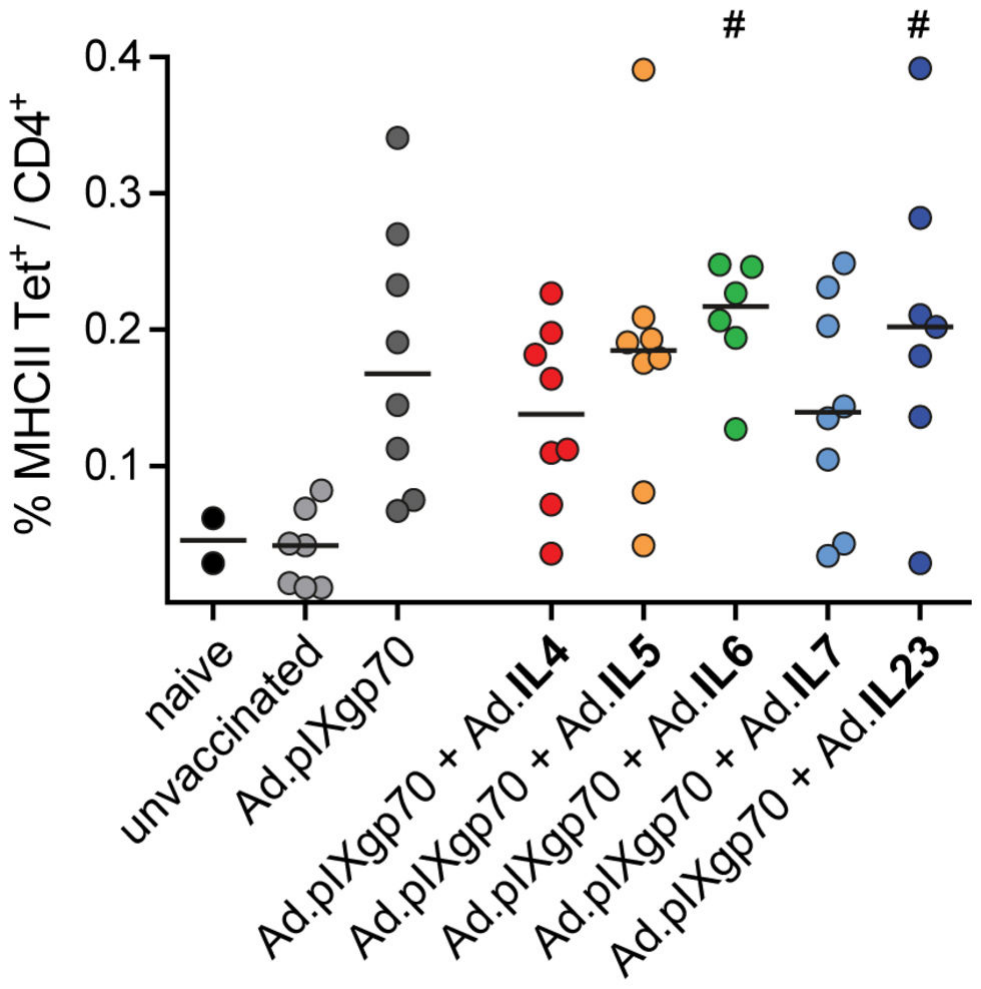
Vaccine-induced F-MuLV Env-specific CD4^+^ T cell responses. CB6F1 mice were immunized twice with Ad.pIXgp70 in combination with adenoviral vectors encoding different interleukins. Three weeks after the second immunization, mice were challenged with 500 SFFU FV. Virus-specific CD4^+^ T cell responses were analyzed 3 days p.i. by tetramer staining of spleen cells. Statistically significant differences (*P* < 0.05; Kruskal-Wallis one-way analysis of variance on ranks with Dunns multiple comparison procedure) compared to unvaccinated mice (#) are indicated. Each dot represents an individual animal, horizontal lines indicate mean values. Data are results of two independent experiments with similar outcome.

Protection from retroviral infection requires complex immune responses including virus-specific CD4^+^ T cells. It was shown before that vaccination with a higher dose of Ad.pIXgp70 alone already resulted in strong CD4^+^ T cell responses [21]. To analyze whether co-immunization with interleukin-encoding vectors further improved the CD4^+^ T cell response, we removed spleens from immunized mice 3 days after FV challenge and virus-specific CD4^+^ T cell responses were quantified by MHC II tetramer staining. We used a lower FV dose of 500 SFFU for this infection as it only served as a boost to make the CD4^+^ T cell response more readily detectable. After immunization with the reduced dose of Ad.pIXgp70 an FV specific CD4^+^ T cell response was detectable, however this was not significantly higher compared to unvaccinated control mice. While the co-application of Ad.IL4, Ad.IL5, or Ad.IL7 did not lead to significant differences in the CD4^+^ T cell response, co-immunization with Ad.pIXgp70 and Ad.IL6 or Ad.IL23 resulted in significantly higher levels of FV-specific CD4^+^ T cells compared to unvaccinated control mice (*P* < 0.05; Figure 4).

## Discussion

The incidence of HIV infection is still a fundamental health problem especially in developing countries. To this day, no protective vaccine has been developed, and while the combined antiretroviral therapy (cART) can effectively reduce viral loads in treated patients over long periods of time, complete virus elimination cannot be achieved by this therapy. Therefore the development of a protective vaccine is a crucial goal in the fight against the HIV pandemic. Previously it has been shown in animal models and also gleaned from results of HIV vaccine trials that an effective anti-retroviral vaccine should elicit broad immune responses conferring antibody- and cell-mediated protection [2,3,6,24], but especially the induction of strong and broadly neutralizing antibody responses by a safe vaccine is a challenging task. In FV infection, vaccination with the adenovirus-based Env gp70 expression/display vector Ad.pIXgp70 resulted in strong protection against FV challenge with high titers of FV-specific binding antibodies [21], and we now set out to further improve this vaccine using interleukins as genetic adjuvants.

The most pronounced effect on vaccination outcome was observed when IL5 or IL23 encoding vectors were used as genetic adjuvants together with the Env gp70 expression/display Ad vector (see Table 1 for a summary of the results). In both cases we found significantly enhanced neutralizing antibody titers after FV challenge infection, which likely mediated the improved control over FV. As IL5 is known to induce the terminal differentiation of activated B cells into antibody-producing cells and to stimulate antibody production by them [14], this is a very plausible finding. IL23 on the other hand has been described as a growth factor for memory T cells and a survival factor for T_H_17 cells and is implicated in promoting cell-mediated autoimmunity [25], however, no direct influence on B cells has been described. When IL23 was used before as genetic adjuvant in DNA-based immunizations of mice against HCV or influenza, improved cellular, but not humoral, immune responses were reported [26,27]. As we saw an improvement of the antibody response, but no major effect on the induction of FV-specific CD4^+^ T cells, our results imply an unknown, direct effect of IL23 on B cells.

**Table 1:** Summary of immunization experiments.

	unvaccinated	Ad.pIXgp70 + Ad.empty	Ad.pIXgp70 + Ad.**IL4**	Ad.pIXgp70 + Ad.**IL5**	Ad.pIXgp70 + Ad.**IL6**	Ad.pIXgp70 + Ad.**IL7**	Ad.pIXgp70 + Ad.**IL23**
spleen weight 21 dpc (gram; mean ± SE)	3,66 ± 0,28	2,20 ± 0,46	2,05 ± 0,42	0,96 ± 0,39	0,91 ± 0,38	2,47 ± 0,50	0,76 ± 0,37
viral load in plasma 10 dpc (FFU / ml; median)	9,99×10^4^	6,35×10^4^	2,97×10^3^	nd	nd	3,80×10^3^	nd
viral load in spleen 21 dpc (SFFU / spleen; median)	1,50×10^7^	1,21×10^6^	3,25×10^4^	1,47×10^3^	nd	4,68×10^4^	3,61×10^2^
FV-specific CD4^+^ T cells 3 dpc (% MHC II Tet**^*+*^** / CD4^+^; mean ± SE)	0,04 ± 0,01	0,18 ± 0,03	0,14 ± 0,02	0,18 ± 0,04	0,21 ± 0,02	0,14 ± 0,03	0,20 ± 0,04
neutralizing antibody titer 10 dpc (median)	4	8	8	64	24	16	48
neutralizing IgG antibody titer 10 dpc (median)	nd	nd	5,15	104	128	20	48

abbreviations: dpc, days post challenge; nd, not detectable

We also saw reduced viral loads after FV challenge when mice had been co-immunized with IL4 or IL6 compared to Ad.pIXgp70 alone, although the effect was less pronounced than that seen for IL5 and IL23. IL4 is the archetypical T_H_2 cytokine, but both IL4 and IL6 have similar properties, stimulating B cell maturation and T_H_2 cell proliferation [13,28]. It is not clear from our results how the slight improvement of control over FV replication after IL4 co-immunization was mediated, as there was no noteworthy change in immune responses. Since the reduction of viral loads was far less pronounced than for some of the other interleukins we tested, IL4 seems a negligible adjuvant candidate for adenovirus-based immunization. In the SIV model it was demonstrated that the vaccination efficacy of live-attenuated SIV could be improved by a nef-deleted SIV vector encoding IL4, and protection correlated with higher antibody titers and higher antibody affinity [29], suggesting that the adjuvant potential of genetic adjuvants may depend on the vector used for immunization. After IL6 co-immunization on the other hand, there was a trend towards higher neutralizing antibodies and an improved CD4^+^ T cell response, which may explain improved control over FV challenge. The fact that adenovirus vector administration itself induces IL6 expression may explain why the effect was not more pronounced [30].

Of the five interleukins tested, IL7 had the least pronounced effect of all, with no significantly changed viral loads at both time points and no significant differences in the immune response. IL7 has an important role in T cell homeostasis [31] and T cell development [16,32,33], promoting proliferation and survival of T cells, as well as promoting B cell maturation in bone marrow and in the lymph node marginal zone [34,35]. The reason for the ineffectiveness of IL7 in adjuvanting our adenovirus-based vaccine is probably inadequate timing of the IL7 expression with regard to T cell promotion as it is not present in the memory phase, and inadequate location with regard to B cell stimulation as IL7 expression is not localized to lymph node marginal zones.

In the study presented here we used only one dose of adenoviral vectors, and equal numbers of vaccine antigen and adjuvant vector particles. We therefore cannot exclude that the adjuvant effect of the interleukin vectors may be stronger for lower vector doses or different vaccine antigen vector – adjuvant vector ratios, including those interleukins that did not show any significant effect on immunization efficacy in the presented study. 

It is important to note that in the groups of mice that had received IL5, IL6, or IL23 as genetic adjuvant, a large number of mice were very strongly protected and no viral load was detectable in the assays performed, even though the mice had received a vaccine that induces only antibodies and CD4^+^ T cells. We could previously show that immunization in the FV model with conventional adenoviral vectors could be improved when we co-applied vectors encoding specific type I interferons or chemokines [19,20], and also here the improvement of vaccine-induced protection was mediated mainly by enhanced induction of FV-specific neutralizing antibodies and CD4^+^ T cells, underlining their important role in anti-retrovirus immunity. However, it was demonstrated before in the FV model that besides CD4^+^ T cell responses and neutralizing antibody responses, CD8^+^ T cells are crucial for complete, sterile protection from FV infection [6]. Protection conferred by vaccination with interleukin- adjuvanted Ad.pIXgp70 vector is very strong despite the absence of a CD8^+^ T cell inducing component, thus by including a vector that induces strong CD8^+^ T cell responses [36] we shall be able to engineer a vector-based vaccine that is similarly effective as a live-attenuated retrovirus and confers complete protection from retrovirus infection. 

Our findings should be transferable to vaccination of humans with adenoviral vectors, against HIV for example, as the functions of interleukins are very homologous between mouse and humans. The adjuvant effect was most apparent after challenge infection in the experiments performed here, which may make it difficult to detect in prophylactic vaccine testing; nonetheless, it may be a valuable tool for therapeutic vaccination. As the changes in the cytokine milieu are only occurring locally, no serious, systemic side effects should be expected from the use of interleukins as genetic adjuvants; none were observed here in the mice co-immunized with interleukin-encoding vectors. The fact that we found increased neutralizing antibody titers but not increased FV-specific CD4^+^ T cell responses makes this approach all the more appealing for HIV vaccine development, as HIV preferably infects HIV-specific CD4^+^ T cells [37] and vaccine-induced HIV-specific CD4^+^ T cells have come under close scrutiny for a potential role in augmented infection rates in non-human primate and human vaccinees in several preclinical and clinical studies [38–40]. Thus, an adjuvant that works directly on B cells without at the same time potentiating CD4^+^ T cell responses is indeed a very desirable tool.

## Conclusions

This study demonstrates an adjuvant effect of murine interleukins in an adenovirus-based vaccination approach against FV infection. While the co-application of Ad.IL4 or Ad.IL7 did not have significant effects on vaccination efficacy, co-immunization of mice with Ad.IL5, Ad.IL6 or Ad.IL23 with Ad.pIXgp70 resulted in improved protection with reduced viral loads and better control over FV-induced disease. For Ad.IL5 and Ad.IL23, this improvement correlated with enhanced neutralizing antibody titers after FV challenge infection, whereas mice co-immunized with Ad.IL6 showed improved virus-specific CD4^+^ T cells. These results show that the co-application of selected interleukin-encoding vectors can be a potent tool to further improve adenovirus-based anti-retroviral vaccination.

## Methods

### Cells and cell culture

The human embryonic kidney cell lines 293A (Invitrogen, Karlsruhe, Germany) and 293T (CRL-11268; American Type Culture Collection, Manassas, VA) were propagated in Dulbecco’s modified Eagle medium with high glucose. A murine fibroblast cell line from *Mus dunni* [41] was maintained in RPMI medium (Invitrogen/Gibco, Karlsruhe, Germany). Cell culture media were supplemented with 10 % fetal bovine serum (Biochrom, Berlin, Germany) and 1 % penicillin/streptomycin (PAA, Pasching, Austria). Cell lines were maintained in a humidified 5 % CO_2_ atmosphere at 37°C.

### Adenoviral vectors

The adenoviral vectors Ad5.pIXgp70 and Ad5F35.pIXgp70 that encode a fusion protein of adenovirus capsid protein pIX and F-MuLV envelope gp70 have been described before [21]. 

For the construction of interleukin-encoding vectors, the coding sequences were amplified from murine RNA by RT-PCR and subcloned into pShuttle plasmid; recombinant Ad5 and Ad5F35 based vectors were obtained by homologous recombination of pShuttle constructs with pAdEasy-1 and pAdEasy-1/F35 [42] and transfection into 293A cells as described before [43]. All interleukin-encoding vectors were purified with the Vivapure AdenoPACK 20 kit and Ad5.pIXgp70 and Ad5F35.pIXgp70 with the Vivapure AdenoPACK 100 kit (Sartorius, Göttingen, Germany). The adenovirus particle concentrations were determined by spectrophotometry as described previously [44] and expressed as viral particles (vp)/ml. The ratio of viral particles to infectious particles was ~30:1 for all viral vector preparations.

Expression levels of the interleukins by the viruses were verified in the supernatants of transfected 293T cells by ELISA (IL5, IL6, IL23: ebioscience, Frankfurt, Germany; IL4: BD biosciences, Heidelberg, Germany; IL7: R&D Systems, Wiesbaden, Germany).

### Mice

Female CB6F1 hybrid mice (BALB/c x C57BL/6 F1; H-2^b/d^ FV1^b/b^ Fv2^r/s^ Rfv3^r/s^) were purchased from Charles River Laboratories (Sulzfeld, Germany). Mice entered experiments when they were between 7 and 9 weeks of age and treated in accordance with the national law and the institutional guidelines of the University Hospital Essen, Germany. The study was approved by the Northrhine-Westphalia State Office for Nature, Environment and Consumer Protection (LANUV NRW) and was carried out on the project license numbers 87-51.04.2010.A063 and 84-02.04.2012.A266 issued by the same state office. 

### Immunization

For immunization against FV, CB6F1 mice were immunized with Ad5- and Ad5F35-based vectors using a heterologous prime-boost immunization protocol with a 28-day interval. 1 x 10^9^ vp of Ad.pIXgp70 vector was mixed with either 1 x 10^9^ vp of interleukin-encoding vectors or a GFP-encoding or empty vector as a control and injected in 100 µl PBS subcutaneously. Prime immunizations were performed with Ad5 vectors and boost immunizations with fiber-chimeric Ad5F35 vectors.

### FV and challenge infection

Uncloned, lactate dehydrogenase-elevating virus (LDV)-free FV stock was obtained from BALB/c mouse spleen cell homogenate (10 % w/v) 14 days p.i. with a B‑tropic, polycythemia-inducing FV complex [45]. CB6F1 mice were infected with 2 500 spleen focus forming units (SFFU) by intravenous injection. The course of infection was determined twice a week by palpation of the spleen of each animal under general anesthesia. Spleen sizes were rated on a scale ranging from 1 (normal spleen size) to 4 (severe splenomegaly) as described previously [46].

When mice were infected with FV for subsequent MHC II tetramer staining, 500 SFFU FV were injected intravenously.

### Viremia Assay

Plasma samples from CB6F1 mice were obtained 10 days p.i. and plasma viral load was determined in a focal infectivity assay [47]. Serial dilutions of plasma were incubated with *M. dunni* cells for 3 days under standard tissue culture conditions. When cells reached ~100 % confluency, they were fixed with ethanol, labelled with F-MuLV Env-specific MAb 720 [48], and then with a horseradish peroxidase (HRP)-conjugated goat anti-mouse antibody (Dako, Glostrup, Denmark). For detection of foci, aminoethylcarbazole (Sigma-Aldrich, Deisenhofen, Germany) was used as HRP substrate. Foci were counted and focus-forming units (FFU) / ml plasma were calculated.

### Infectious center assay

FV-infected animals were sacrificed 21 days p.i. by cervical dislocation, spleens were removed and weighed and single-cell suspensions were prepared. Serial dilutions of isolated spleen cells were added to *M. dunni* cells and incubated under standard tissue culture conditions for 3 days and then fixed and stained as described for the viremia assay. Foci were counted and infectious centers (IC) / spleen were calculated.

### Binding antibody ELISA

To detect F-MuLV-specific binding antibodies, Nunc Maxi-Sorp ELISA (Nunc, Langenselbold, Germany) plates were coated with 0.5 µg whole F-MuLV antigen per well, blocked with 10 % fetal calf serum in PBS and incubated with serum dilutions. For detection a polyclonal goat anti-mouse HRP-coupled anti IgG (dianova, Hamburg, Germany), HRP anti-mouse IgG1 (Becton Dickinson, Heidelberg, Germany) or HRP-conjugated goat anti-mouse IgG2a (Bethyl/Biomol, Hamburg, Germany) and as substrate tetramethylbenzidine (TMB+, Dako, Glostrup, Denmark) were used. Sera were positive if the optical density at 450 nm was 3-fold higher than that measured for sera of naive mice.

### Complement-dependent F-MuLV-neutralizing antibody assay

For analysis of neutralizing antibodies, plasma samples were inactivated by incubating at 56°C for 30 minutes, and serially diluted with PBS. For the analysis of neutralizing antibodies of IgG type, heat-inactivated plasma samples were mixed with an equal volume of 0.2 M β-mercapto-ethanol, incubated for 30 minutes at 37°C, and then serially diluted with PBS containing 0.01 M β-mercapto-ethanol. Plasma dilutions were mixed with purified F-MuLV and guinea pig complement (Sigma-Aldrich, München, Germay) and incubated for 1 h at 37°C. Then the samples were added to *M. dunni* cells which were plated in 24-well plates the day before at a density of 7.5 x 10^3^ cells per well. Cells were incubated to ~100 % confluency under standard tissue culture conditions and fixed and stained as described for the viremia assay. Foci were counted and dilutions which resulted in a reduction of foci number by 75 % or more were considered neutralizing.

### Tetramer staining of F-MuLV-specific CD4^+^ T cells

Three days p.i. spleens of CB6F1 mice were removed and single-cell suspensions were prepared. For staining of FV-specific CD4^+^ T cells, cells were labelled with an allophycocyanin (APC)-coupled major histocompatibility complex (MHC) class II tetramer (containing the I-Ab-restricted F-MuLV Env epitope EPLTSLTPRCNTAWNRLKL [49]; kindly provided by the MHC Tetramer Core Facility of the National Institutes of Health, National Institute of Allergy and Infectious Disease, Atlanta, GA), phycoerythrin (PE)-anti CD4 and fluorescein isothiocyanate (FITC)-anti CD11b (BD biosciences, Heidelberg, Germany), and propidium iodide (eBioscience, Frankfurt, Germany) for exclusion of dead cells. Acquisition of data was performed on a flow cytometer (FACS LSR II, Becton Dickinson, Mountain View, CA) and for analysis FACSDiva (Becton Dickinson, Mountain View, CA) and FlowJo (version 7.6.5, Tree Star, Ashland, OR) software were used.

### Statistical analyses

Statistical analyses were performed using the software GraphPad Prism 5 (GraphPad Software, La Jolla, CA), testing with the Kruskal-Wallis one-way analysis of variance on ranks and Dunns multiple comparison procedure.

## Supporting Information

Figure S1 **Schematic represenation of the vaccine vectors.** (A) The gp70 expression-display vector is deleted of E1, E3, and the native pIX sequence, and encodes a fusion protein of pIX and F-MuLV gp70 as a transgene in the E1 region. (B) Interleukin-encoding vectors are deleted of E1 and E3 and contain the coding sequences for murine IL4, IL5, IL6, IL7, or IL23. Restriction endonucleases used for cloning of the interleukin sequences are indicated (* IL6 was sub-cloned by ligation of a *Bgl*II and a *Bam*HI fragment). ITR, inverted terminal repeat; Ψ, packaging signal; CMV-IE, cytomegalovirus immediate early promoter; pA, polyadenylation signal.
(EPS)

Figure S2 **Experimental outline.** (A) Mice were immunized twice with Ad.pIXgp70 in combination with adenoviral vectors encoding specific interleukins. Mice were first immunized with Ad5-based vectors and 4 weeks later with Ad5F35 vectors. 3 weeks after the second immunization, mice were infected with 2500 SFFU FV. Antibody titers were analyzed 3 weeks after the first immunization, 2 weeks after the second immunization, and 10 days after FV challenge infection. Viral load in plasma was analyzed 10 days after FV challenge, viral load in spleens was analyzed 3 weeks after FV challenge. (B) For analysis of CD4^+^ T cell responses, mice were immunized as above, and infected with a reduced dose of 500 SFFU FV 3 weeks after the second immunization. CD4^+^ T cells were characterized by MHC II tetramer staining 3 days after FV challenge.
(EPS)

Figure S3 **Protection from FV challenge after immunization with interleukin-encoding vectors alone.** CB6F1 mice were immunized twice with adenoviral vectors encoding IL5, IL6 or IL23. Mice were immunized first with Ad5 vectors and after 4 weeks, mice were immunized with Ad5F35 vectors. Three weeks after the second immunization, mice were challenged with 2500 SFFU FV and spleens were removed and weighed 3 weeks after challenge infection (A). Viral load in plasma was analyzed 10 days after FV challenge (B) and viral load in spleens was analyzed 21 days after FV challenge (C). Each dot represents an individual mouse, horizontal lines indicate mean (A) or median values (B,C). The dashed lines indicate the detection limits.
(EPS)
